# Research Trends and Future Perspectives in Marine Biomimicking Robotics

**DOI:** 10.3390/s21113778

**Published:** 2021-05-29

**Authors:** Jacopo Aguzzi, Corrado Costa, Marcello Calisti, Valerio Funari, Sergio Stefanni, Roberto Danovaro, Helena I. Gomes, Fabrizio Vecchi, Lewis R. Dartnell, Peter Weiss, Kathrin Nowak, Damianos Chatzievangelou, Simone Marini

**Affiliations:** 1 Department of Renewable Marine Resources, Instituto de Ciencias del Mar (ICM-CSIC), 08003 Barcelona, Spain; 2 Stazione Zoologica Anton Dohrn (SZN), 80122 Naples, Italy; valerio.funari@bo.ismar.cnr.it (V.F.); sergio.stefanni@szn.it (S.S.); r.danovaro@univpm.it (R.D.); fabrizio.vecchi@szn.it (F.V.); 3 Centro di Ricerca Ingegneria e Trasformazioni Agroalimentari, Consiglio per la Ricerca in Agricoltura e l’Analisi dell’Economia Agraria (CREA), 00015 Rome, Italy; 4 The BioRobotics Institute, Scuola Superiore Sant’Anna (SSAA), 56127 Pisa, Italy; marcello.calisti@santannapisa.it; 5 Lincoln Institute for Agri-food Technology (LIAT), University of Lincoln, Lincoln LN6 7TS, UK; 6 Consiglio Nazionale delle Ricerche (CNR), Istituto di Scienze Marine (ISMAR), 40129 Bologna, Italy; 7 Department of Life and Environmental Science, Università Politecnica delle Marche, 60121 Ancona, Italy; 8 Faculty of Engineering, University of Nottingham, Nottingham NG7 2RD, UK; Helena.Gomes1@nottingham.ac.uk; 9 School of Life Sciences, University of Westminster, London W1W 6UW, UK; lewis.dartnell@gmail.com; 10 Spartan Space, 13275 Marseille, France; p.weiss@spartan-space.com; 11 Compagnie Maritime d’Expertises (COMEX), 13275 Marseille, France; k.nowak@comex.fr; 12 Department of Physics and Earth Sciences, Jacobs University, 28759 Bremen, Germany; damchatzi@gmail.com; 13 Consiglio Nazionale delle Ricerche (CNR), Istituto di Scienze Marine (ISMAR), 19032 La Spezia, Italy; simone.marini@sp.ismar.cnr.it

**Keywords:** marine biomimetics, bibliographic statistics, energy provision, biomaterials, locomotor designs, optimal energy consumption

## Abstract

Mechatronic and soft robotics are taking inspiration from the animal kingdom to create new high-performance robots. Here, we focused on marine biomimetic research and used innovative bibliographic statistics tools, to highlight established and emerging knowledge domains. A total of 6980 scientific publications retrieved from the Scopus database (1950–2020), evidencing a sharp research increase in 2003–2004. Clustering analysis of countries collaborations showed two major Asian-North America and European clusters. Three significant areas appeared: (i) energy provision, whose advancement mainly relies on microbial fuel cells, (ii) biomaterials for not yet fully operational soft-robotic solutions; and finally (iii), design and control, chiefly oriented to locomotor designs. In this scenario, marine biomimicking robotics still lacks solutions for the long-lasting energy provision, which presently hinders operation autonomy. In the research environment, identifying natural processes by which living organisms obtain energy is thus urgent to sustain energy-demanding tasks while, at the same time, the natural designs must increasingly inform to optimize energy consumption.

## 1. Introduction

We are experiencing a “robotics revolution” [1] under the paradigm of mechatronics, which combines mechanical, computer, telecommunications, and control engineering, all multidisciplinarily integrated for the creation of autonomously operating robots [2]. An entirely new class of flying, walking, and swimming robots is currently under development [3]. Unprecedented robotic capabilities are being implemented, likely contributing to the next industrial era in worldwide economies, including the marine sector [4].

The term biomimicking refers to the use of Nature as inspiration for the design and development of technological systems, as a promising methodology to generate a new breed of autonomous machines [5,6]. In the last decades, marine mechatronic is taking inspiration from several animal phyla to create mobile robots [7,8,9]. To give a few examples, salamanders [10], insects [11], birds [12], swimming robo-tuna, -salmon, -manta, and -jellyfish (respectively by [13,14,15,16] and their walking counterparts as the 6-legged Crabster CR200 and Silver 2, or robo-lobsters [17,18], have been created. Additionally, SCUBA droids [19], remotely controlled through immersive 3D visual interfaces [20], are biomimicking human functionalities to be used for remote in situ operations [21].

Marine robotic development is subjected to energy provision and traditional tools for that task are batteries, chemical fuel cells and (super)capacitors [22]. New biologically-inspired reactors such as microbial fuel cells (MFC) and other bioelectrochemical systems (BES) are being proposed as reservoirs to powering robotic platforms [23,24]. MFC and BES are biologically catalyzed electrochemical systems, in which microorganisms perform the oxidation and reduction of raw chemical substrates (as nutrients) at the electrodes, to generate energy for motion, sensing and computation [25,26].

In this scenario, environmental sciences benefit from such a development in robotics, including the biomimicking sector, to provide new data picturing the complex changes in natural and human-impacted ecosystems [27]. Robotics provide a generous contribution to a breakthrough in “Earth System Science”, embracing the idea that geosphere, hydrosphere, atmosphere, and anthroposphere are interconnected into global change frameworks [28,29]. Therefore, new robotic solutions are increasingly capable of operating without human supervision across ecosystems and under virtually any habitat condition [30] in a cooperative and intercommunicating mode [31,32,33,34]. Robots are being used for a wide range of agricultural, industrial, and broad environmental activities, e.g., [35,36,37], including forefront deep-sea and extra-terrestrial operational scenarios, e.g., [31,38,39,40]). For example, in the oceanic domains, robots’ data-gathering provides long-lasting time series on species presence and their abundances and environmental data in geographically and three-dimensionally extended water column-seabed scenarios [21,31,40]. This highly integrated spatiotemporal monitoring is finally opening new possibilities to understand how environmental processes shape life responses in cause-effects relationships, e.g., [41,42].

Here, we used innovative bibliographic statistics tools for information content metrics and mapping as the traditional survey approaches, based on some studies selected from the literature, often do not fully represent the state of the art of a specific scientific domain. They produce a bias, caused by the arbitrary choice of the discussed studies, that do not offer a comprehensive background for theory development [43,44]. As a consequence, many surveys are based on the systematic study of the literature, across different scientific domains, like building engineering [45], business and marketing [46], waste management [47] and medicine [48]. The statistical approach proposed in this work is based on [49,50,51] and aimed to highlight the most promising bio-inspired robotics marine research areas, identifying temporal trends, some established and emerging research targets, and the degree of international cooperation. We focused on marine biomimetics for three main reasons: (i) ocean exploration is challenging and the exploration and monitoring require the development of innovative platforms technologies, also fulfilling the needs for space research, e.g., [52,53]; (ii) underwater organisms show unique adaptations to extreme conditions, which could be of inspiration for entirely novel robotic developments; and finally, (iii) marine organisms have dynamic reactions when they perceive the presence of current platforms [54,55,56,57,58,59] as remotely operated vehicles (ROVs), autonomous underwater vehicles (AUVs) and internet operated vehicles (IoVs) as crawlers, so that the biomimetic development will improve the exploration capability, to picture biodiversity to an extent never attained before.

## 2. Materials and Methods

### 2.1. Database Search

The Scopus database was consulted on 27 August 2020 and used to retrieve bibliographic records related to biomimicking for 1950–2020. The search was restricted to scientific publications written in English, avoiding biomedicine and agricultural oriented results.

To identify relevant publications, the following keywords were used in the combined fields of title, abstract and keywords (per publication): TITLE-ABS-KEY (bioinspired OR bioinspiration OR “biologically-inspired” OR biophysics OR bionics OR biomimicking OR biomimics OR biomimetics OR biomimic OR biomimetic OR bio-mimicking OR bio-inspired OR “microbial fuel cell” OR “bio fuel cell” OR bioenergy OR bio-energy) AND (marine OR sea OR underwater OR water OR ocean OR exocean OR exo-ocean OR astrobiology OR moon OR moons OR “planets” OR deep-sea OR deepsea OR acid-mine-drainage) AND (design OR cybernetics OR robot OR mechatronic OR vehicle OR engineering OR mechanical OR technologies OR materials) AND NOT (agricultural OR agriculture OR tumour OR orthopaedic OR fouling OR pharmacology OR medicine OR tumor OR antibiotics OR lubricants OR lubricant OR dentist OR dentistry OR dental OR dentin OR surgical OR bone OR surgery OR disease OR clinic OR clinical OR “biomedical device” OR biomedicine OR solution OR endoprosthesis OR trauma OR orthopaedic OR surgery OR “in vitro” OR drug OR anticoagulant OR inflammatory OR “oil spill” OR saliva OR salivary OR therapy OR healing OR wound OR europium OR urban OR megacity OR megacities OR child OR children OR dietary OR nurse OR “medical imaging” OR tomography OR “magnetic resonance” OR x-ray OR nutrition OR pregnancy OR “body water” OR nursing OR infancy OR “comparative study”) AND PUBYEAR >1950.

The publications resulting from the query were validated in order to avoid false positives, especially in the cases were the singular and plural forms of a term might bear a different meaning.

### 2.2. Bibliometric Mapping and Clustering

This study is based on the latest advancements in science mapping, to provide the most comprehensive and systematic review of bio-inspired robotics marine research. While a traditional narrative review may base its findings on 50–200 studies subjectively chosen by a researcher, this study uses the entire Elsevier Scopus database with a statistically formal approach that has slimmed down a list of 6980 articles on since 1950. The results are robust and reproducible, which infers the reliability of the offered methodology. This is a modern and quantitative way of reviewing a subject area [60]. A general quantitative description of the bibliographic records was conducted, based on all the publications returned by the query. A word cloud of country names with different font sizes as a proxy of the number of publications (based on co-authors affiliations), was constructed. The number of publications for a single country was computed considering all the co-authorships of each published item.

Bibliometric maps were created on retrieved publications, using the VOSviewer software version 1.6.5.0. The software was specifically developed for creating, visualizing and exploring science’s bibliometric maps [61]. A term-map, also called co-word map, is a two-dimensional representation of a research field. The larger the number of publications in which two terms occurring in titles, abstracts and keywords, co-occur, the stronger the terms are related [49,50,51,62]. In a term-map, strongly related terms are located close to each other, and the weaker the relationship is between terms, the bigger the distance is between them [63]. For this purpose, VOSviewer uses a clustering technique that can be seen as a kind of weighted variant-of-modularity-based clustering [64,65]. After the clustering, terms that belong to the same domain of knowledge have the same color. Only terms occurring at least eight times were extracted from the retrieved publications and a subset of more relevant labels was displayed in the maps to avoid text overlapping.

Before starting with the analysis in VOSviewer, a thesaurus was created to ensure consistency for different term spelling and synonyms. As an example, “biomimetic”, “biomimetic approach”, “biomimicry” or “biomimetic system” were listed as synonymous with “biomimetic”. Another example was “energy”, “energy production”, “energy source” or “power” substituted with “energy” or “microbial fuel cells”, “microbial fuel cells technology”, “MFC”, “MFCs”, “MFC system”, and “MFC technology” all named as “microbial fuel cells”. A final example was represented by “fish robot”, “robot fish” or “robotic fish” collectively named as “robotic fish”. For the scope of this paper, we prepared three types of maps: (i) a term-clustering map; (ii) a term-year map, and (iii) a term-citation map.

### 2.3. The Term-Year Map

For the term-year map, the color of a term indicates the average publication year of all the publications in which the term occurs. As for the previous term-citation map, we used colors that range from blue (mean year term presence 2011 or earlier), through yellow (2014), to red (2016 or later). Therefore, blue terms mainly occur in older publications, while red terms are in more recent publications.

### 2.4. The Term-Citation Map

In the term-citation map, the color of a term is determined by the average citation impact of the publications where the term occurs. The number of citations of each publication is divided by the average number of citations of all publications appearing in the same year, to avoid biases related to the age of a publication (older publications are expected to be more cited). This produces a publication’s normalized citation score range from 0 to 2.5. In the map, the colors are assigned according to the score, ranging from blue (average score of 0) to green (average score of 1.25) to red (average score of 2.5). Therefore, a blue (cold) or red (hot) term indicates low and high average citation impacts, respectively [66].

### 2.5. Statistical Analyses

The mean values of the average publication year or term-citation map for each group obtained by the cluster analysis was tested on the null hypothesis of the same median (Kruskal-Wallis Test) being the distributions not normal (Shapiro-Wilk Test) and homoscedastic (Levene Test); Mann-Whitney pairwise comparisons and Bonferroni corrected Post Hoc Test were applied. Basic statistics were performed using the software PAST [67].

## 3. Results

### 3.1. Journals, Subject Categories and Countries

A total of 6980 publications were retrieved from the Scopus database encompassing 70 years (i.e., 1950–2020). The selected pool of scientific references includes 63.1% of them are research papers, while the remaining 36.9% are distributed in this way: 23.7% of conference papers, 8.9% reviews, 3.1% book chapters, and 1.3% is of miscellaneous origin (e.g., letters to editors, short communications, notes). The query results showed that a total of 146 journals published at least eight or more papers in the field of biomimicking.

The top ten journals, in terms of the number of papers, are reported in Table 1, and the top three are the following: *Bioresource Technology* (1991; *n* = 167; 2.39%) *Bioinspiration and Biomimetics* (2006; *n* = 147; 2.11%) and *Proceedings of SPIE the International Society for Optical Engineering* (1963; *n* = 107; 1.53%). Early biomimicry papers were published in general engineering/electronics journals, and then as the field developed, more papers were published in dedicated biomimetics journals as they came into existence. The complete list of publications numbers per subject category is reported in Table A1, Appendix A.

**Figure 1:**
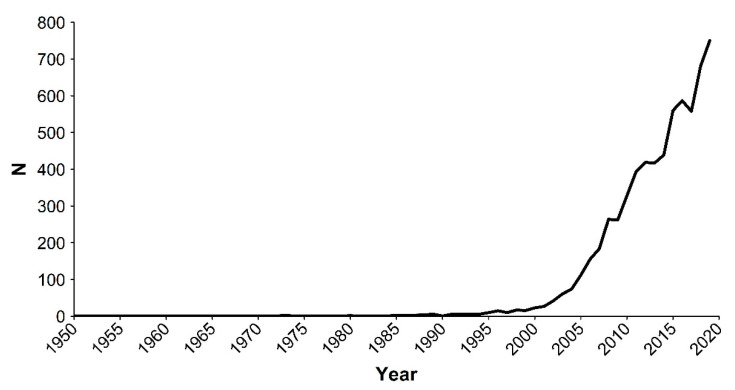
Temporal trend in biomimicking publications. Publications in the time interval 1950 to 2020, with *n* being the number of papers issued per year. One should note that data for publications of 2020 are not represented since underestimated (i.e., the search was conducted on August 2020, and not all published items for that year were included in the Scopus database).

In Figure 1, the number of biomimicking publications produced within the time interval 1950 to 2020 is shown to highlight the temporal trends. Before 2004, the total number of publications was low (350; 5.0%), then it drastically and continuously increased with a sharper slope until 2019, with data for 2020 being incomplete (as per the date of the query).

As represented by the grouping of the papers in the top subject categories, the publication is reported in Table 2. Only the first ten categories are reported, as they account for 90.4% of the total available papers.

**Figure 2:**
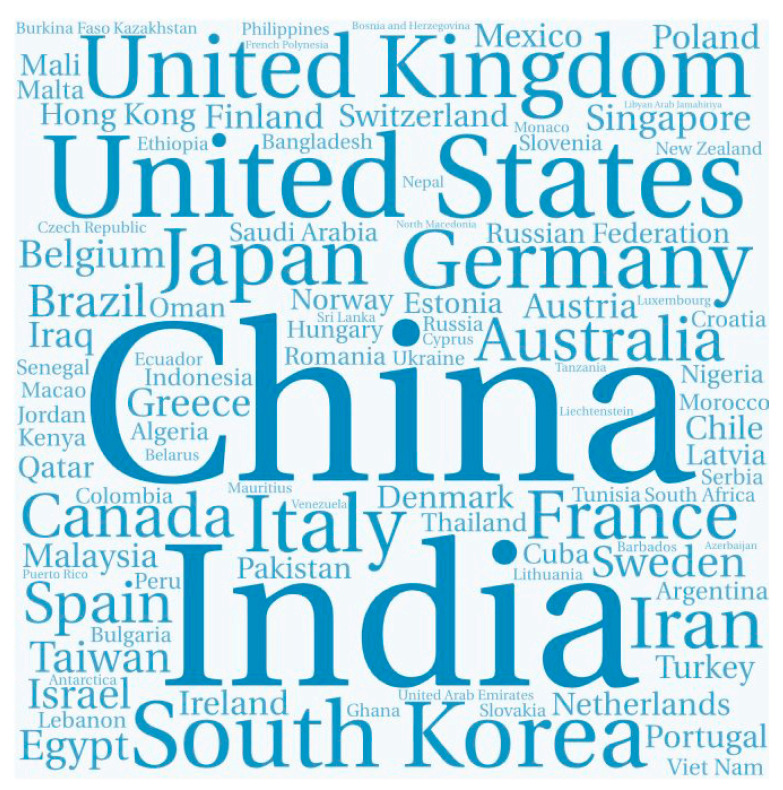
Word cloud chart of the authors’ affiliations. The different font sizes of the countries are proportional to the number of publications. The position of the country in this visual map is randomized.

Authors that published at least one paper focused on biomimicking, were affiliated to a total of 100 countries (see Table A2 for the complete country list). Figure 2 shows the word cloud chart representing the countries (in blue text), where the larger font size represents a higher number of publications: China (24.1%, *n* = 2183), United States (18.9%, *n* = 1714), United Kingdom (5.0%, *n* = 451), India (4.2%, *n* = 384), Germany (4.0%, *n* = 364), Japan (3.7%, *n* = 339), South Korea (3.2%, *n* = 294), Italy (2.7%, *n* = 241), and Australia (2.6%, *n* = 234).

**Figure 3:**
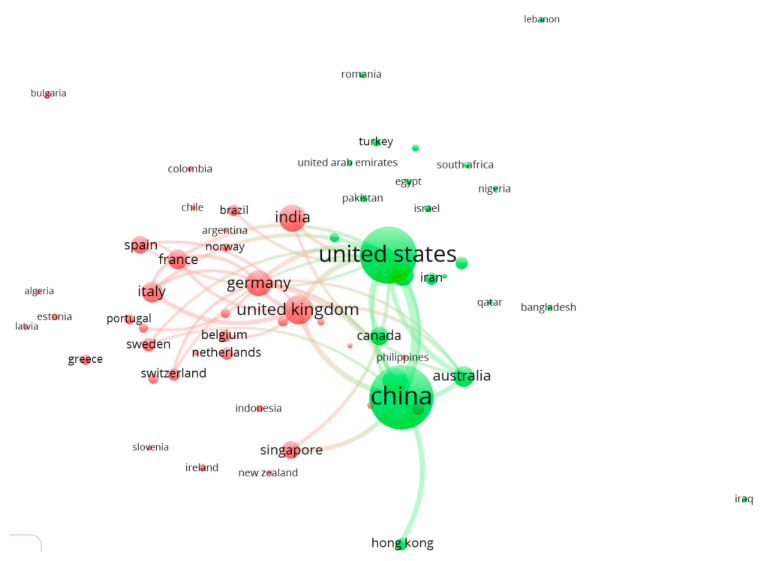
Clustering of the countries’ collaborations and their reciprocal links. Clustering is based on the authors’ addresses. Different colors represent different countries’ clusters (only the first 50 linkages in terms of relevance were illustrated).

Figure 3 displays the countries’ color-coded collaborative clustering, with two different major groups: Asia-North America (USA and Canada; green) *versus* Europe (red). The partnership between USA and China (and Hong Kong) is evidenced by the association of both with Canada and Commonwealth countries, as Australia (but not New Zealand). The United Kingdom and Germany are the core for the European cluster in association with Spain, France, and Italy, plus Asian countries of Singapore and India. However, the patchy distribution of the collaborations (same color but no connections) of satellite countries around both red and green clusters indicates occasional, temporally sparse and non-sustained collaborations (i.e., below the 50 linkages).

### 3.2. The Term-Clustering Map Identifying Major Research Areas

**Figure 4:**
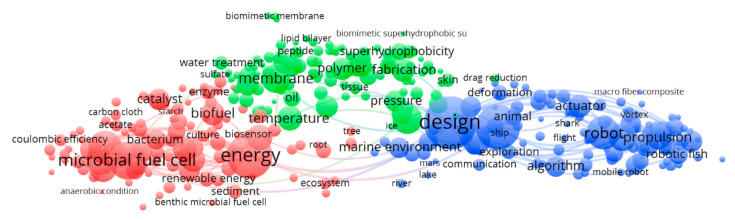
Term-map analysis based on biomimicking publications. Different colors represent the terms belonging to different clusters. Terms are represented by a circle (node), whose diameter and the label size represent the number of publications, where that term appears. Top 100 linkages were represented.

The outputs of term-clustering map analysis are presented in Figure 4. Three major color-coded groups are identified based on the differential level of interconnection between each cluster and among the clusters.

**Figure A1:**
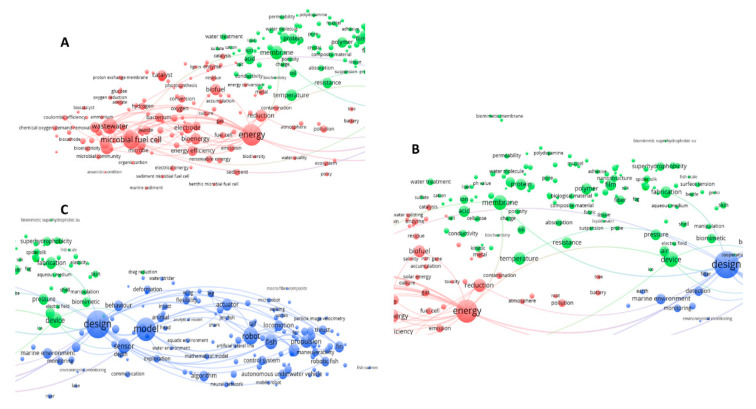
The enlarged view of the 3 clusters (**A**–**C**) from the term-map analysis.

In Figure A1, the clusters are enlarged, to show the connections among their elements better. The terms grouped in the three clusters can be summarized and hence named as energy provision (red), biomaterials (green), and design and control (blue).

According to the most relevant terms, the red cluster identifies the biologically-based energy solutions (see also Figure A1A). The four top-ranked terms concerning the averaged normalized citation were: “energy” (9635), “microbial fuel cell” (7398), “wastewater” (5663), and “electricity” (4103). The terms refer to processes based on “bio-energy” production (“enzyme”, “biocathode”, “biocatalyst”, “biofuel”), centred on bacteria (“bacterium”, “culture” and “microbe”) and the utilization of microbial communities’ metabolism, to sustain that energy provision (e.g., “microbial fuel cell”, “bioenergy” and “energy” in general), with special attention to aerobic and anaerobic solutions (i.e., “chemical oxygen demand”, “oxygen reduction” and “anaerobic condition”). That cluster is also related to energy provision based on electricity production (i.e., “biocathode”, “bioelectricity”, “electrical energy”, “electrode”, “battery”, “coulombic efficiency”, and “conversion”), including new renewable ways for its production (i.e., “gas”, “hydrogen”, “photosynthesis”). Industrial procedures (e.g., “wastewater” and “water quality”, as well as “waste”, “contamination”, “residue”, “metal” and “emission”) are also included. Operational factors affecting microbial energy provision by “microbial fuel cells” in different marine deployment areas are also evidenced (i.e., “marine sediment”, “sediment microbial fuel cells” and “benthic microbial fuel cells”), as is “ecosystem” in general.

The biomaterials (green) cluster (see also Figure A1B) has a relevant aspect concerning the development of nature-inspired composites for both energy provision (green items close to the red cluster; see above) and new robotic functionalities (green items close to the blue cluster; see below). The analysis related to the four top-ranked terms for this cluster concerning the averaged normalized citation shows the following: (i) “device” (4007); (ii) “membrane” (3953); (iii) “temperature” (2528); and (iv) “fabrication” (2280). In the left part of this cluster, there are terms mainly related to energy provision as, e.g., for “charge”, “conductivity”, “salt”, “ion”, “cation”, “sulphate”, “pH value”, “acid” and “temperature”. On the central and right part, terms are related to new biomimicking composites (e.g., “polymer”, “fibre”, “composite material”, “biomimetic membrane”, “biomimetic superhydrophobic surfaces”) at different size scales (e.g., “nanostructure”, “film”), cable to operate under different conditions (e.g., “resistance”, “aqueous medium”, “pressure”, “permeability”, “superhydrophobicity”). Interestingly, animal “biomimetic” approaches appear within this cluster side at the level of specific solutions as “mussel”, “gecko”, “beetle”, for membrane functionalities related to, e.g., adhesion (i.e., “adhesive”). In this cluster right half there are also items as “tissue” (i.e., “skin” and “shell”), or “polymer” whose engineering is bio-inspired (e.g., “silk”, “spider silk”, “cellulose”), along with “fabrication” methods (e.g., “lipid” and “lipid bilayer” or “biological material” as “peptide” and “protein”) for “permeability” (with “pore” and “porosity”), “absorption” and “suspension” functionalities in “liquid water” “fog”, “aqueous medium” or “ice”.

The design and control (blue) cluster (see also Figure A1C) has terms dealing with robotic “design” and “model” within all types of platforms (including the biomimicking ones), dedicated to the “environmental monitoring” as well as the “exploration” of the “marine environment”, “river”, “lake” and all “aquatic environments” in general, including also space research (“Mars”). The analysis related to the four top-ranked terms concerning the averaged normalized citation indicates the following: “design” (10857), “model” (7452), “robot” (4625) and “fish” (3698). This cluster encompasses the system control, actuators and computational features (i.e., “neural network”, “genetic algorithm”, and “algorithm”). This cluster shows a high level of elements interconnection at the level of prototype and platforms design for autonomous robotic animal-inspired solutions (“animal” and “robotic fish”, exemplified by “shark”, “jellyfish”, and “squid”). The method developments for underwater motility (“thrust” and “propulsion”) likely discriminate underwater vehicles from robots adopting more biomimicking solutions for locomotion. Advanced motility systems (e.g., “walking” and “swimming”) by biomimicked appendages (e.g., “wing”, “leg” and “fin”) appear related to hydrodynamic simulations (e.g., “particle image velocimetry”, “analytical model” and “mathematical model”), with a higher relevance in term of interconnection than “autonomous underwater vehicle”.

### 3.3. Publication Trends: Years and Citation Rate

**Figure 5:**
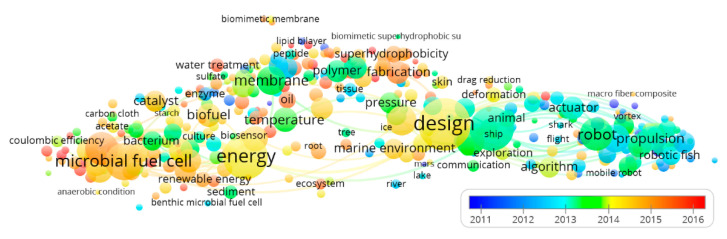
Term-year map based on biomimicking publications. The scale represented the earlier (blue) or more recent (red) years when the term appeared. Terms are represented by a circle (node whose diameter and label size represent the number of publications where the term occurs. Top 100 linkages were represented.

**Figure 6:**
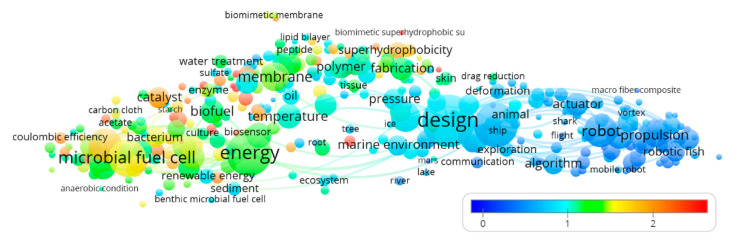
Term-citation map (average normalized citation) based on biomimicking publications. The scale represented the earlier (blue) or more recent (red) years when the term appeared. Terms are represented by a circle (node whose diameter and label size represent the number of publications where the term appears. Top 100 linkages were represented.

The term-year map based on “biomimicking” papers (Figure 5) presents a temporal trend where energy provision (red cluster of Figure 4) at the level of fuel cells and design and control (blue cluster of Figure 4) at the level of the design itself shows the most recent field of research. Also, the different levels of elements interconnection for each of the three recognized clusters (see Figure 4) are a proxy of the robotic field’s maturation level. Control and propulsion methods appear as the oldest (within the blue cluster), biomimetic designs have an intermediate age, and membranes engineering are the newest (along with bioenergy sustained solutions). The different maturation level of the research scenario within each of the three clusters is also visible for citations ranges (Figure 6). The four top-ranked terms for the red cluster (energy provision) in terms of averaged citation rate are: “ionic liquid” (3.72), “biochar” (2.92), “water splitting” (2.91), and “battery” (2.41). These four items for the green cluster (biomaterials) are: “salt” (4.01), “biomimetic superhydrophobic surface” (3.00), “gecko” (83.56), and “metal ion” (2.84). The four top-ranked terms for the blue cluster (design and control) are: “filter” (1.77), “gravity” (1.07), “environmental monitoring” (1.07), and “insect” (1.00).

Considering the mean values of the average publication year for the three groups obtained by the cluster analysis (see Figure 4), the red cluster (energy provision) showed a more recent mean value (2014.4), followed by the green one (biomaterials; 2014.3) and the blue one (design and control; 2013.3). Considering the mean values of the average normalized citation rate for the three groups obtained by the cluster analysis (see Figure 4), the red cluster (energy provision) showed a higher normalized citation rate (1.46), followed by the green one (biomaterials; 1.30) and the blue one (design and control; 0.56). For both the publication years and citation trend, the goodness of the data was tested on the null hypothesis of the same median, being the distributions not normal (Shapiro-Wilk test; *p* < 0.05) and homoscedastic (Levene Test; *p* > 0.05); Kruskal-Wallis rejected the null hypothesis of equality of median. Mann-Whitney pairwise comparisons Bonferroni corrected post-hoc test showed a significant difference (*p* < 0.001) between all the clusters.

## 4. Discussion

Our results pointed out that the main emerging fields of marine biomimicking robotic research are the provision of energy via microbial fuel cells, biomaterials for the development of not yet fully operational soft-robotic solutions, and a more classic bioinspired design and control, associated with a multiplication of locomotion solutions. In this scenario, one central aspect is the lack of long-lasting energy provision, which to date conditions robots’ operation autonomy. Energy generation could represent a solution via internal systems in a homeostatic interaction with the surrounding environment. For this reason, marine bio-inspiration is becoming increasingly oriented toward the identification of metabolic processes by which living organisms obtain energy from their environment and the natural designs allowing the optimization of energy consumption.

### 4.1. The Temporal Trends in Biomimicking Robotics Research

Biomimicking robotic research has poor development prior to 2000 (i.e., only 123 out of 6980 are publications related to this subject prior that year, constituting 1.76% of the total retrieved with the query). From 2003–2004 onwards, a sharp increase occurred, which is still lasting today without signs of saturation (see Figure 1). Since 2000, there has been an increasing interest in developing technological products for the maritime and offshore industries and science, with marine robotics enabling the execution of increasingly complex industrial and military missions [37]. After 2004, U.S. military research [68] and the industrial innovation in Europe and Asia [69] broadly stimulated general robotic research. At the same time, in 2004, the U.S. Commission on Ocean Policy evaluated that a large part of the U.S. marine infrastructure was obsolete, which advocated for research toward its renewal [70].

More recent research developments are related to efforts for energy provision and storage (see next section). For example, the advent of microbial fuel cells (MFC) contributed to the publication peak in 2016 [71] and added new perspectives for energy supply, especially after the appearance of the miniaturized supercapacitive MFC [72]. Also, membrane separation technology and biosorbents are some of the branches that developed quickly after the 2000s due to the interest in purification and recovery of strategic metals from complex matrices, e.g., [73,74]. The latest advances in electrodialysis have provided clues on the control of electron transfer during the treatment of different materials using MFC, e.g., [75]. In the past five years, using biocatalysts in electrode materials led to significant improvements in efficiency compared to abiotic anode [76]. Bioelectrochemical systems (BES), anode surface modifications with nanomaterials, and bacterial gene modifications are becoming prevalent approaches to improve MFC performances [77]. In this last decade, biofuel production technologies contributed to energy provision research and new nanomaterials or nanotechnologies [22,77], including actions taken to improve memory storage capacity in robotic applications for data exchange [23,78].

Another reason justifying the recent growth in robotic biomimicry research is space exploration. Such an exploration is not only oriented to Mars, but it actually includes the sub-surface oceans of Europa and Enceladus and other icy moons as equivalents to deep-sea terrestrial environments [38,53,79,80]. Proposed applications plan to use vectors to carry biomimicking probes for their delivery into specific terrestrial, atmospheric and aquatic environments, orienting biomimicking research toward the design of the structural hardware and toward the emulation of specific behavioral aspects. Platforms operative cooperation is biomimicked considering the example of sociality in insects [81,82,83]. For instance, behavioral biomimicking is being used as a reference to build swarms of travelling platforms with differential distribution of tasks in the atmosphere of Mars (e.g., the flying bees for Mars [84]).

### 4.2. Biomimicking Energy Provision

Our results on the biomimicking design for energy provision in the underwater environment (red cluster; see Figure 4) evidenced its relationships with the conditioning needs of locomotion functionalities. That biomimicking is only partially developed at the level of metabolism-inspired functionalities, with microbial fuel cells (MFCs) becoming a hot topic in the recent literature developments (see the previous section and Figure 5).

Using MFCs to produce and distribute energy is provided by the electrolytic mediators through an external circuit. So this design is far to be seen as an equivalent of metabolism in living organisms since it is not diffused throughout the robotic structures. However, robotic systems powered by MFCs are usually termed ‘gastrobots’, which means robots with a stomach [23,85]. So, organ-like structures are being conceived to provide energy, exploiting metabolic reactions to power, e.g., motility. Such an energy system often requires a pulsed or opportunistic behavior to perform tasks and sub-tasks because the energy supply cannot support a continuous mode operation [23,78].

Biochemical applications simulating metabolism in robots have been developed but not yet coupled into biomechanics designs. Under this aspect, the challenge is linked to the power density that microbial fuel cells can output: computation, locomotion and interaction, especially in harsh environments, are extremely energy demanding for the bigger robots, and novel approaches are required to reduce such energy demands [86]. The energy density (per unit of mass) is still insignificant compared to standard fuels like gasoline or Li-ion batteries, so a combination of different technologies is still required for the autonomy of robotic systems [23,78]. However, the electricity produced by the microbial fuel cell can be further stored in a capacitor for burst-mode power delivery [23]. Also, a microbial fuel cell can be transformed into a microbial electrolysis cell (MEC) [87] to produce ancillary biofuel (hydrogen energy) eventually. This type of cell may be the biotic substitute for traditional batteries to supply the required voltage. Combining these two types of microbial cells may be suited for almost entirely self-sustained motility of small weights [77].

To achieve sufficient energy performances and maintain control over physio-chemical variations (homeostasis) is challenging, such that few results have been transferred into the industry [22]. The main limiting factor is the energy requirement. Nano-research is vital to lighten the mechanical structure and “organs”, but when it comes to working with bare microbial fuel cells, volumetric energy density is an essential parameter for mobile applications [22] due to the limited spaces available inside the devices. Even if a higher gravimetric energy density than a metal-based fuel cell is generated, smaller volumetric energy density will limit the application range of those cells alone [22,88]. From this viewpoint, MFC relying on organic cathode materials with a low mass density might demonstrate limited performances [24].

In this scenario, the metabolism-inspired functionalities for energy provision sustaining marine platforms autonomous operability has still poor connectivity within the overall energy provision research scenario (e.g., “benthic fuel cells” and “sediment fuel cells” appearing as separated in the red cluster; see Figure A1A). The U.S. Naval Research Laboratory has used sediment MFC to harvest energy from the seafloor for low-power consuming applications like meteorological buoys [89]. Biosensors powered by MFC have drawn increasing interest due to their sustainability and cost-effectiveness, with several applications for water quality monitoring [90,91], which can be used in aquatic robots. MFC-based biosensors are being used to detect metals such as copper, chromium, and zinc, as well as organic compounds [91], although there is a need to improve their sensitivity, as they have high detection thresholds.

In the next future, the design of efficient and effective metabolic engineering approaches will increasingly benefit from artificial intelligence (AI) methodologies [92]. AI can picture retrobiosynthesis (a reverse engineering-like approach, where metabolism can be disentangled in terms of originating reactions and their relationship) approaches, highlighting key reaction rules present in biological systems [93]. Moreover, machine learning techniques are used to incorporate genomic data for predicting the optimal feed substrate of MFCs [94], while machine learning can be used for modelling and controlling the temperature inside those cells [95].

### 4.3. Biomimicking Materials for Robotics

Biomaterials biomimicking is the replication of biological processes at the molecular level with human-made materials [96]. In this context, our results indicated that the terms in this cluster (green; see Figure 4) have an identity related to the creation of new solutions at scales ranging from macro to micro. Biomimicry is used to solve conflictual engineering problems across very different multi-functional materials size ranges to merge flexibility and resistance [97,98]. The existence of that cluster in our analysis as separated from that of the design and control (see the next section) indicates that robots planning and construction is still conceived along two independent lines: one using traditional materials in large sizes mechanically-designed platforms and one looking for new functionalities at the level of membrane and surfaces for soft robotic frontline applications.

The implementation of bio-inspired materials at nanoscales is a research field parallel but still poorly connected to robotic mechanical design. Microfabrication of membranes is not yet at a sufficient level of technological development to have substantially impacted the published literature we analyzed and therefore does not appear prominently within the green cluster. The rightmost terms (“gecko”, “beetle”, etc.), which may point toward the design of bioinspired systems (blue cluster), resulted instead primarily for the micro properties of the bioinspired mechanism, e.g., adhesion [99], or the properties of fabrication process of the animals, e.g., spider silk [100,101]. Another example is represented by the study of marine organisms’ biomacromolecules (e.g., hyaluronic acid, chitin and chitosan, peptides, collagen, enzymes, algal polysaccharides). Reef fish mucus, marine adhesives and structural coloration are biomimicked to recreate adhesives, collagen, or coating-antifouling materials [102,103,104].

Therefore, this cluster belongs to the bottom-up approach to biomimicking: the quest here is to create new materials, technologies and devices, which replicate specific biological solutions. Those solutions have to be intended as the building blocks for further general applications (e.g., gecko adhesion for vertical locomotor functionalities). Moreover, technology can rarely grant a perfect mimicking of the natural solution. In those cases, the phenomena are abstracted at a high level, eventually instantiating concrete products with still low diffusion in robotics applications [98].

### 4.4. Biomimicking Design and Control

The design and control cluster (blue; see Figure 4) highlighted that most robotic applications mimic innovative automated body motions in associations with the morphology of different types of aquatic animals. That quest for improved locomotion highlights the need to move beyond the state of the art of traditional robotic platforms [2,105]. The result is creating biomimetic underwater drones with thrusters-independent swimming capability, as a step forward compared to the more conventional designs such as autonomous underwater vehicles (AUVs) and gliders [106]. This is confirmed in our results (see Figure A1C) by the relevance of terms we identified in our survey, all related to animal morphological models (i.e., “jellyfish”, “shark”, “fish”, “squid”, and even “insect”) with different levels of association with mobility (“manoeuvrability”, “propulsion”, “thrust”, “mobile robot”, etc.). Also, terms like “actuators” and “sensors” and their linksappear related to locomotion effectiveness rather than generic functionality.

Interestingly, our research revealed how mobility functionalities are prioritized over manipulation (i.e., robot having appendages to interact with objects). The “manipulation” term appears only on the far-right side of the biomaterial cluster (see previous section and Figure A1B,C). By considering the vast amount of research in robot manipulation and robotic grippers [107,108], and the huge bioinspired efforts carried out in creating artificial hand prosthesis [109,110,111], it is surprising that marine robotics manipulation is underrepresented, and this may be a promising area for future developments, especially concerning bio-ecological and industrial applications.

Two reasons could lie beyond that situation. First, locomotion efforts are still far from being completed and satisfactory, and therefore no attention is directed toward underwater bioinspired manipulation. This appears less probable due to the high request for innovative grippers and manipulators employed in the underwater domain [112,113]. A second reason seems more realistic: if we consider the usual bioinspired path, peculiar, smart, or novel biological solutions should be identified by life scientists and eventually proposed to engineers. We believe this path is presently underexploited, and that additional research into animals/systems that could be used as a reference for novel bioinspired grippers and arms would be profitable. The oil industry historically developed underwater robotic manipulators to work on metal components. Still, for biological applications such as sampling delicate/fragile species, innovative solutions in soft robotics are required, e.g., [114,115].

AI is not apparent in the design and control cluster, since its development is very recent. An AI-related key question emerging from this cluster is which combination of sensorial technologies allows full autonomy in robotic functionalities. A promising research field is within the hardware devices for underwater vision. It is clear that marine animals evolved several solutions to adapt their vision system to the underwater environment [116], and several attempts were performed into bio-inspired underwater vision. Examples span from the biomimicking of vision to guide vehicles as the use of underwater light polarization-sensitivity (by shrimps [117]), to the neuro-model driving anguilliform-swimming devices (by lamprey [116]), and celestial compass-based approaches (by ants [118]). Also, non-light oriented approaches were implemented as the use of turbulence and pressure changes to reconstructs 3D panoramas (i.e., seal whiskers [119]) or the sensing of weak electric fields near objects (muddy water fishes [120]).

Other applications of AI are related to swarm intelligence [121,122], neural simulation [123,124], and evolutionary computing [125] for controlling fleets of vehicles. Similarly, neural networks are often used to detect and localize underwater objects [126,127], fins sensors can inform the design of control systems of fin-driven robots [128], while bio-inspired algorithms are applied for localizing odour or chemical sources by underwater robots [129].

### 4.5. Limitations of Our Bibliographic Meta-Analysis

There are some limitations in the term-map analysis regarding both the production and the interpretation of results. There are mainly two kinds of limitations: those generated by the data and those imposed by the map. For the first aspect, during the creation of the bibliometric dataset, the record’s availability could have been somehow limited mainly due to the arbitrary keywords’ choice for the primary search. Also, regarding this aspect, synonyms and homonyms represent another kind of problem that could arise. Although this problem was solved with an accurate manual thesaurus polishing by merging the synonymous terms (see the methodological section “Bibliometric Mapping and Clustering”), more advanced approaches can be used based on the formal conceptualization of the marine science domain [130]. For example, the Semantic Web for Earth and Environmental Terminology (SWEET) ontology [131] and the Natural Environment Research Council (NERC; https://vocab.nerc.ac.uk/ assessed on 28 May 2021) vocabulary could be used for the automated merging of the terms resulting from the abstract, titles and keyword analysis. Similarly, natural processing language techniques can also be used to identify the most relevant terms, on which the map is built up [132,133].

For the second aspect, the interpretation of a bibliometric map is not always straightforward. A term-map represents a simplified version of reality, leading to loss of information and a partial representation of the investigated field [134]. VOSviewer generates such an inevitable loss of information due to the two-dimensional representation of the terms in a Euclidean space. We are aware that those mapping and clustering procedures rely on dissimilar design principles and conventions. However, the VOS mapping technique and the weighted and parameterized variant of modularity-based clustering can both be derived from the same underlying principle [64]. This justified our choice of a unified approach to mapping and clustering bibliometric networks to identify sub-fields or specific sub-topics [135]. Despite these limitations, depending on researcher errors and bibliometric mapping constraints, term-map analysis represents a valuable tool to support experts to improve their knowledge on a specific domain [135].

## Figures and Tables

**Table 1:** The top ten journals, in terms of year of official foundation, number of papers (*n*) published from 1950 to 2020 on biomimicking along with their relative frequency (%).

Rank	Journal	Year	*n*	%
1	*Bioresource Technology*	1991	167	2.39
2	*Bioinspiration and Biomimetics*	2006	147	2.11
3	*Proceedings of SPIE the International Society for Optical Engineering*	1963	107	1.53
4	*ACS Applied Materials and Interfaces*	2009	105	1.50
5	*Journal of Bionic Engineering*	2004	80	1.15
6	*Langmuir*	1985	70	1.00
7	*Environmental Science and Technology*	1967	48	0.69
8	*Advanced Materials*	1989	46	0.66
9	*Biomass and Bioenergy*	1991	46	0.66
10	*Water Science and Technology*	1969	45	0.64

**Table 2:** Number (*n*) of publications per each subject category as a different discipline.

Discipline	*n*
Engineering	2985
Materials Science	1707
Chemistry	1402
Chemical Engineering	1290
Environmental Science	1259
Computer Science	1193
Biochemistry, Genetics and Molecular Biology	1152
Physics and Astronomy	1109
Energy	896
Mathematics	525
Others	1438

## Data Availability

The EndNote file containing the database of the publication extracted is available online at https://doi.org/10.6084/m9.figshare.13337327 (Figshare repository, accessed on 28 May 2021).

## Appendix A

**Table A1:** Number of publications (*n*) per the complete list of subject categories as different disciplines (see Table 2).

Subject Area	*n*
Engineering	2985
Materials Science	1707
Chemistry	1402
Chemical Engineering	1290
Environmental Science	1259
Computer Science	1193
Biochemistry, Genetics and Molecular Biology	1152
Physics and Astronomy	1109
Energy	896
Mathematics	525
Agricultural and Biological Sciences	420
Earth and Planetary Sciences	285
Immunology and Microbiology	174
Multidisciplinary	146
Medicine	133
Social Sciences	79
Business, Management and Accounting	53
Neuroscience	35
Pharmacology, Toxicology and Pharmaceutics	24
Economics, Econometrics and Finance	22
Arts and Humanities	19
Health Professions	18
Decision Sciences	17
Psychology	7
Veterinary	4
Dentistry	1
Nursing	1

**Table A2:** Complete list of countries and their total number of issued scientific papers (*n*) as well as their relative percentage.

Country	*n*	%	Country	*n*	%	Country	*n*	%	Country	*n*	%
China	2183	26.573	Mexico	36	0.438	Ethiopia	6	0.073	Monaco	1	0.012
United States	1714	20.864	Israel	34	0.414	Lithuania	6	0.073	Nepal	1	0.012
United Kingdom	451	5.490	Saudi Arabia	34	0.414	Oman	5	0.061	North Macedonia	1	0.012
India	384	4.674	Pakistan	32	0.390	Croatia	4	0.049	Peru	1	0.012
Germany	364	4.431	Ireland	31	0.377	Kenya	4	0.049	Sri Lanka	1	0.012
Japan	339	4.127	Estonia	30	0.365	Senegal	4	0.049	Tanzania	1	0.012
South Korea	294	3.579	Indonesia	29	0.353	Slovakia	4	0.049	Venezuela	1	0.012
Italy	241	2.934	Thailand	28	0.341	Tunisia	4	0.049			
Australia	234	2.848	Egypt	26	0.316	Ecuador	3	0.037			
France	210	2.556	South Africa	26	0.316	Luxembourg	3	0.037			
Canada	187	2.276	Romania	21	0.256	Macao	3	0.037			
Spain	175	2.130	Argentina	20	0.243	Mauritius	3	0.037			
Singapore	173	2.106	Czech Republic	20	0.243	Puerto Rico	3	0.037			
Sweden	98	1.193	New Zealand	20	0.243	Cuba	2	0.024			
Taiwan	92	1.120	Viet Nam	20	0.243	Jordan	2	0.024			
Hong Kong	86	1.047	Hungary	19	0.231	Kazakhstan	2	0.024			
Belgium	84	1.023	Bangladesh	16	0.195	Russia	2	0.024			
Iran	84	1.023	Chile	16	0.195	Serbia	2	0.024			
Netherlands	84	1.023	Nigeria	14	0.170	Antarctica	1	0.012			
Switzerland	84	1.023	Colombia	11	0.134	Azerbaijan	1	0.012			
Malaysia	82	0.998	Iraq	11	0.134	Barbados	1	0.012			
Brazil	80	0.974	Philippines	11	0.134	Belarus	1	0.012			
Denmark	63	0.767	Qatar	11	0.134	Bosnia and Herzegovina	1	0.012			
Greece	61	0.743	United Arab Emirates	10	0.122	Burkina Faso	1	0.012			
Poland	61	0.743	Bulgaria	9	0.110	Cyprus	1	0.012			
Portugal	57	0.694	Latvia	9	0.110	French Polynesia	1	0.012			
Russian Federation	57	0.694	Lebanon	9	0.110	Ghana	1	0.012			
Austria	55	0.670	Algeria	8	0.097	Libyan Arab Jamahiriya	1	0.012			
Finland	52	0.633	Morocco	8	0.097	Liechtenstein	1	0.012			
Turkey	44	0.536	Slovenia	8	0.097	Mali	1	0.012			
Norway	42	0.511	Ukraine	7	0.085	Malta	1	0.012			

## References

[1] Van Wynsberghe A., Donhauser J. (2018). The dawning of the ethics of environmental robots. Sci. Eng. Ethics.

[2] Yang G.Z., Bellingham J., Dupont P.E., Fischer P., Floridi L., Full R., Jacobstein N., Kumar V., McNutt M., Merrifield R. (2018). The grand challenges of Science Robotics. Sci. Robot..

[3] Kim S., Laschi C., Trimmer B. (2013). Soft robotics: A bioinspired evolution in robotics. Trends Biotechnol..

[4] Degnarian N., McCauley D. 12 robots that could make (or break) the oceans. World Economic Forum. https://www.weforum.org/agenda/2016/09/12-cutting-edge-technologies-that-could-save-our-oceans?utm_content=bufferf4c29&utm_medium=social&utm_source=plus.google.com&utm_campaign=buffer.

[5] Fu K., Moreno D., Yang M., Wood K.L. (2014). Bio-inspired design: An overview investigating open questions from the broader field of design-by-analogy. J. Mech. Des..

[6] Snell-Rood E. (2016). Interdisciplinarity: Bring biologists into biomimetics. Nature.

[7] Atkeson C.G., Hale J.G., Pollick F., Riley M., Kotosaka S., Schaul S., Shibata T., Tevatia G., Ude A., Vijayakumar S. (2000). Using humanoid robots to study human behavior. IEEE Intell. Syst..

[8] Bhushan B. (2009). Biomimetics: Lessons from nature-an overview. Philos. Trans. R. Soc. A.

[9] Calisti M., Picardi G., Laschi C. (2017). Fundamentals of soft robot locomotion. J. R. Soc. Interface.

[10] Ijspeert A.J., Crespi A., Ryczko D., Cabelguen J.M. (2007). From swimming to walking with a salamander robot driven by a spinal cord model. Science.

[11] Wood R.J. (2008). The first take-off of a biologically inspired at-scale robotic insect. IEEE Trans. Robot..

[12] Fei F., Tu Z., Zhang J., Deng X. Learning extreme hummingbird manoeuvres on flapping wing robots. Proceedings of the 2019 International Conference on Robotics and Automation ICRA.

[13] Anderson J.M., Chhabra N.K. (2002). Manoeuvring and stability performance of a robotic tuna. Integr. Comp. Biol..

[14] Ikeda M., Hikasa S., Watanabe A., Nagai I. (2014). Motion analysis of a manta robot for underwater exploration by propulsive experiments and the design of central pattern generator. Int. J. Autom. Technol..

[15] McColgan J., McGookin E.W. Coordination of a school of robotic fish using nearest neighbour principles. Proceedings of the IEEE OCEANS 2014.

[16] Villanueva A., Bressers S., Tadesse Y., Priya S., Bar-Cohen Y., Wallmersperger T. (2009). Jellyfish inspired underwater unmanned vehicle. Electroactive Polymer Actuators and Devices EAPAD 2009, Proceedings of SPIE SMART STRUCTURES AND MATERIALS + NONDESTRUCTIVE EVALUATION AND HEALTH MONITORING, San Diego, CA, USA, 8–12 March 2009.

[17] Hood E. (2004). RoboLobsters: The beauty of biomimetics. Environ. Health Perspect..

[18] Kim J.Y., Jun B.H. (2014). Design of six-legged walking robot, Little Crabster for underwater walking and operation. Adv. Robot..

[19] Carey B. Maiden voyage of Stanford’s humanoid robotic diver recovers treasures from King Louis XIV’s wrecked flagship. Stanford News. https://news.stanford.edu/2016/04/27/robotic-diver-recovers-treasures.

[20] Khatib O., Yeh X., Brantner G., Soe B., Kim B., Ganguly S., Stuart H., Wang S., Cutkosky M., Edsinger A. (2016). Ocean-One: A robotic avatar for oceanic discovery. IEEE Robot. Autom. Mag..

[21] Danovaro R., Fanelli E., Aguzzi J., Billett D., Carugati L., Corinaldesi C., Dell’Anno A., Gjerde K., Jamieson A.J., Kark S. (2020). Ecological variables for developing a global deep-ocean monitoring and conservation strategy. Nat. Ecol. Evol..

[22] Ue M., Sakaushi K., Uosaki K. (2020). Basic knowledge in battery research bridging the gap between academia and industry. Mater. Horiz..

[23] Ieropoulos I.A., Greenman J., Melhuish C., Horsfield I. (2012). Microbial fuel cells for robotics: Energy autonomy through artificial symbiosis. ChemSusChem.

[24] Schon T.B., McAllister B.T., Li P.F., Seferos D.S. (2016). The rise of organic electrode materials for energy storage. Chem. Soc. Rev..

[25] El Mekawy A., Srikanth S., Bajracharya S., Hegab H.M., Nigam P.S., Singh A., Mohan S.V., Pant D. (2015). Food and agricultural wastes as substrates for Bio-Electrochemical System (BES): The synchronized recovery of sustainable energy and waste treatment. Food Res. Int..

[26] Rabaey K., Angenent L., Schröder U., Keller J. (2009). Bio-Electrochemical Systems: From Extracellular Electron Transfer to Biotechnological Application.

[27] Hart J.K., Martinez K. (2006). Environmental sensor networks: A revolution in the earth system science?. Earth-Sci. Rev..

[28] Jacobson M., Charlson R., Rodhe H., Orians G. (2000). Earth System Science 72: From Biogeochemical Cycles to Global Changes.

[29] Reid W.V., Chen D., Goldfarb L., Hackmann H., Lee Y.T., Mokhele K., Ostrom E., Raivio K., Rockström J., Schellnhuber H.J. (2010). Earth system science for global sustainability: Grand challenges. Science.

[30] Bayat B., Crasta N., Crespi A., Pascoal A.M., Ijspeert A. (2017). Environmental monitoring using autonomous vehicles: A survey of recent searching techniques. Curr. Opin. Biotechnol..

[31] Aguzzi J., Chatzievangelou D., Marini S., Fanelli E., Danovaro R., Flögel S., Lebris N., Juanes F., De Leo F., Del Rio J. (2019). New high-tech interactive and flexible networks for the future monitoring of deep-sea ecosystems. Environ. Sci. Technol..

[32] Duarte M., Gomes J., Costa V., Rodrigues T., Silva F., Lobo V., Monteiro M., Oliveira S.M., Christensen A.L. Application of swarm robotics systems to marine environmental monitoring. Proceedings of the IEEE OCEANS 2016.

[33] Scilimati V., Petitti A., Boccadoro P., Colella R., Di Paola D., Milella A., Grieco L.A. (2017). Industrial Internet of things at work: A case study analysis in robotic-aided environmental monitoring. IET Wirel. Sens. Syst..

[34] Vasilijević A., Nađ Đ., Mandić F., Mišković N., Vukić Z. (2017). Coordinated navigation of surface and underwater marine robotic vehicles for ocean sampling and environmental monitoring. IEEE/ASME Trans. Mechatron..

[35] Batth R.S., Nayyar A., Nagpal A. Internet Of robotic Things: Driving intelligent robotics of future concept, architecture, applications and technologies. Proceedings of the 2018 4th International Conference on Computing Sciences ICCS.

[36] Shukla A., Karki H. (2016). Application of robotics in onshore oil and gas industry–A review Part I. Robot. Auton. Syst..

[37] Zereik E., Bibuli M., Mišković N., Ridao P., Pascoal A. (2018). Challenges and future trends in marine robotics. Ann. Rev. Control.

[38] Dachwald B., Ulamec S., Postberg F., Sohl F., De Vera J.P., Waldman C., Lorenz R.D., Zacny K.A., Hellard H., Biele J. (2020). Key technologies and instrumentation for subsurface exploration of ocean worlds. Space Sci. Rev..

[39] Gao J., Chien S. (2017). Review on space robotics: Toward top-level science through space exploration. Sci. Robot..

[40] Rountree R.A., Aguzzi J., Marini S., Fanelli E., De Leo C.F., Del Rio J., Juanes F., Hawkins S.J., Allcock A.L., Bates A.E., Evans A.J., Firth L.B., McQuaid C.D., Russell B.D., Smith I.P., Swearer S.E., Todd P.A. (2020). Towards an optimal design for ecosystem-level ocean observatories. Oceanography and Marine Biology: An Annual Review.

[41] Aguzzi J., Chatzievangelou D., Francescangeli M., Marini S., Bonofiglio F., Del Río J., Danovaro R. (2020). The hierarchic treatment of marine ecological information from spatial networks of benthic platforms. Sensors.

[42] Dunbabin M., Marques L. (2012). Robots for environmental monitoring: Significant advancements and applications. IEEE Robot. Autom. Mag..

[43] Cobo M.J., López-Herrera A.G., Herrera-Viedma E., Herrera F. (2011). Science mapping software tools: Review, analysis, and cooperative study among tools. J. Am. Soc. Inf. Sci. Technol..

[44] Linnenluecke M.K., Marrone M., Singh A.K. (2020). Conducting systematic literature reviews and bibliometric analyses. Aust. J. Manag..

[45] Ammad S., Alaloul W.S., Saad S., Qureshi A.H. (2021). Personal protective equipment (PPE) usage in construction projects: A scientometric approach. J. Build. Eng..

[46] Valenzuela L.M., Merigó J.M., Johnston W.J., Nicolas C., Jaramillo J.F. (2017). Thirty years of the Journal of Business & Industrial Marketing: A bibliometric analysis. J. Bus. Ind. Mark..

[47] Jin R., Yuan H., Chen Q. (2019). Science mapping approach to assisting the review of construction and demolition waste management research published between 2009 and 2018. Resour. Conserv. Recycl..

[48] Kim M.C., Nam S., Wang F., Zhu Y. (2020). Mapping scientific landscapes in UMLS research: A scientometric review. J. Am. Medic. Inform. Assoc..

[49] Pallottino F., Biocca M., Nardi P., Figorilli S., Menesatti P., Costa C. (2018). Science mapping approach to analyse the research evolution on precision agriculture: World, EU and Italian situation. Precis. Agric..

[50] Costa C., Schurr U., Loreto F., Menesatti P., Carpentier S. (2019). Plant phenotyping research trends, a science mapping approach. Front. Plant Sci..

[51] Costa C., Fanelli E., Marini S., Danovaro R., Aguzzi J. (2020). Global deep-sea biodiversity research trends highlighted by science mapping approach. Front. Mar. Sci..

[52] Jacobstein N., Bellingham J., Yang G.Z. (2017). Robotics for space and marine sciences. Sci. Robot..

[53] Aguzzi J., Flexas M.M., Flögel S., Lo Jacono C., Tagherlini M., Costa C., Marini S., Bahamon N., Martini S., Fanelli E. (2020). Exo-oceans exploration with deep-sea sensor and platform technologies. Astrobiology.

[54] Mitson R.B., Knudsen H.P. (2003). Causes and effects of underwater noise on fish abundance estimation. Aquat. Living Resour..

[55] Nedwell J.R., Lovell J., Turnpenny A.W. (2005). Experimental validation of a species-specific behavioral impact metric for underwater noise. J. Acoust. Soc. Am..

[56] Holmes J.D., Carey W.M., Lynch J.F. (2010). An overview of unmanned underwater vehicle noise in the low to mid frequencies bands. J. Acoust. Soc. Am..

[57] Nichols T.A., Anderson T.W., Širović A. (2015). Intermittent noise induces physiological stress in a coastal marine fish. PLoS ONE.

[58] Edmonds N.J., Firmin C.J., Goldsmith D., Faulkner R.C., Wood D.T. (2016). A review of crustacean sensitivity to high amplitude underwater noise: Data needs for effective risk assessment in relation to UK commercial species. Mar. Pollut. Bull..

[59] Picardi G., Borrelli C., Sarti A., Chimienti G., Calisti M. (2020). A minimal metric for the characterization of acoustic noise emitted by underwater vehicles. Sensors.

[60] Klarin A. (2019). Mapping product and service innovation: A bibliometric analysis and a typology. Technol. Forecast. Soc. Chang..

[61] Van Eck N.J., Waltman L. (2010). Software survey: VOSviewer, a computer program for bibliometric mapping. Scientometrics.

[62] Van Eck N.J., Waltman L. Text mining and visualization using VOSviewer. ArXiv Preprint. https://arxiv.org/abs/1109.2058.

[63] Van Eck N.J., Waltman L., Ding Y., Rousseau R., Wolfram D. (2014). Visualizing bibliometric networks. Measuring Scholarly Impact: Methods and practice.

[64] Waltman L., Van Eck N.J., Noyons E.C.M. (2010). A unified approach to mapping and clustering of bibliometric networks. J. Informetr..

[65] Waltman L., Van Eck N.J. (2013). A smart local moving algorithm for large-scale modularity-based community detection. Eur. Phys. J. B.

[66] Van Eck N.J., Waltman L., Van Raan A.F.J., Klautz J.M.R., Peul W.C. (2013). Citation analysis may severely underestimate the impact of clinical research as compared to basic research. PLoS ONE.

[67] Hammer Ø., Harper D.A.T., Ryan P.D. (2001). PAST: Paleontological statistics software package for education and data analysis. Palaeontol. Electron..

[68] Singer P.W. (2011). Wired for War: The Robotics Revolution and Conflicts in the Twenty-First Century.

[69] United Nations Economic Commission for Europe-UNECE and International Federation of Robotics-IFR (2005). World Robotics 2005—Statistics, Market Analysis, Forecasts, Case Studies and Profitability of Robot Investment.

[70] National Research Council-NRC (2011). Critical Infrastructure for Ocean Research and Societal Needs in 2030.

[71] Santoro C., Arbizzani C., Erable B., Ieropoulos I. (2017). Microbial fuel cells: From fundamentals to applications. A review. J. Power Sour..

[72] Soavi F., Bettini L.G., Piseri P., Milani C., Santoro C., Atanassov P., Arbizzani C. (2016). Miniaturized supercapacitors: Key materials and structures towards autonomous and sustainable devices and systems. J. Power Sour..

[73] Chauhan G., Jadhao P.R., Pant K.K., Nigam K.D.P. (2018). Novel technologies and conventional processes for recovery of metals from waste electrical and electronic equipment: Challenges and opportunities—A review. J. Environ. Chem. Eng..

[74] Won S.W., Kotte P., Wei W., Lim A., Yun Y.S. (2014). Biosorbents for recovery of precious metals. Bioresour. Technol..

[75] Gomes H.I., Funari V., Dinelli E., Soavi F. (2020). Enhanced electrodialytic bioleaching of fly ashes of municipal solid waste incineration for metal recovery. Electrochim. Acta.

[76] Kumar R., Singh L., Zularisam A.W. (2016). Exoelectrogens: Recent advances in molecular drivers involved in extracellular electron transfer and strategies used to improve it for microbial fuel cell applications. Renew. Sustain. Energy Rev..

[77] Kumar R., Singh L., Zularisam A.W., Hai F.I. (2018). Microbial fuel cell is emerging as a versatile technology: A review on its possible applications, challenges and strategies to improve the performances. Int. J. Energy Res..

[78] Melhuish C., Ieropoulos I., Greenman J., Horsfield I. (2006). Energetically autonomous robots: Food for thought. Auton. Robots.

[79] Menon C., Broschart M., Lan N. Biomimetic and robotics for space application: Challenges and emerging technologies. Proceedings of the IEEE International Conference on Robotics and Automation (ICRA)—Workshop on Biomimetic Robotics.

[80] Soderlund K.M., Kalousová K., Buffo J.J., Glein C.R., Goodman J.C., Mitri G., Patterson G.W., Postberg F., Rovira-Navarro M., Rückriemen T. (2020). Ice-Ocean exchange processes in the Jovian and Saturnian satellites. Space Sci. Rev..

[81] Hunt E. The Social Animals That Are Inspiring New Behaviors for Robot Swarms. The Conversation. https://theconversation.com/the-social-animals-that-are-inspiring-new-behaviors-for-robot-swarms-113584.

[82] Romano D., Donati E., Benelli G., Stefanini C. (2019). A review on animal–robot interaction: From bio-hybrid organisms to mixed societies. Biol. Cybern..

[83] Webster-Wood V.A., Akkus O., Gurkan U.A., Chiel H.J., Quinn R.D. (2017). Organismal engineering: Toward a robotic taxonomic key for devices using organic materials. Sci. Robot..

[84] Bluman J.E., Kang C.K., Landrum D.B., Fahimi F., Mesmer B. Marsbee—Can a bee fly on mars?. Proceedings of the 55th American Institute of Aeronautics and Astronautics (AIAA) Aerospace Sciences Meeting 2017-0328.

[85] Wilkinson S. (2000). Gastrobots—Benefits and challenges of microbial fuel cells in foodpowered robot applications. Auton. Robots.

[86] Kumar S.S., Kumar V., Kumar R., Malyan S.K., Pugazhendhi A. (2019). Microbial fuel cells as a sustainable platform technology for bioenergy, biosensing, environmental monitoring, and other low power device applications. Fuel.

[87] Hong L., Grot S., Logan B.E. (2005). Electrochemically assisted microbial production of hydrogen from acetate. Environ. Sci. Technol..

[88] Logan B.E. (2010). Scaling up microbial fuel cells and other bioelectrochemical systems. Appl. Microbiol. Biotechnol..

[89] Tender L.M., Gray S.A., Groveman E., Lowy D.A., Kauffman P., Melhado J., Tyce R.C., Flynn D., Petrecca R., Dobarro J. (2008). The first demonstration of a microbial fuel cell as a viable power supply: Powering a meteorological buoy. J. Power Sources.

[90] Chouler J., Cruz-Izquierdo Á., Rengaraj S., Scott J.L., Di Lorenzo M. (2018). A screen-printed paper microbial fuel cell biosensor for detection of toxic compounds in water. Biosens. Bioelectron..

[91] Zhou T., Han H., Liu P., Xiong J., Tian F., Li X. (2017). Microbial fuels cell-based biosensor for toxicity detection: A review. Sensors.

[92] Dasgupta A., Chowdhury N., De R.K. (2020). Metabolic pathway engineering: Perspectives and applications. Comput. Methods Programs Biomed..

[93] Kim G.B., Kim W.J., Kim H.U., Lee S.Y. (2020). Machine learning applications in systems metabolic engineering. Curr. Opin. Biotechnol..

[94] Cai W., Lesnik K.L., Wade M.J., Heidrich E.S., Wang Y., Liu H. (2019). Incorporating microbial community data with machine learning techniques to predict feed substrates in microbial fuel cells. Biosens. Bioelectron..

[95] Yewale A., Methekar R., Agrawal S. (2020). Multiple model-based control of multi variable continuous microbial fuel cell (CMFC) using machine learning approaches. Comput. Chem. Eng..

[96] Wanieck K., Fayemi P.E., Maranzana N., Zollfrank C., Jacobs S. (2017). Biomimetics and its tools. Bioinspir. Biomim. Nanobiomater..

[97] Vincent J.F., Mann D.L. (2002). Systematic technology transfers from biology to engineering. Philos. Trans. R. Soc. A.

[98] Vincent J.F.V., Bogatyreva I.A., Bogatyrev N.R., Bowyer A., Pahl A.K. (2006). Biomimetics: Its practice and theory. J. R. Soc. Interface.

[99] Lee H., Lee B.P., Messersmith P.B. (2007). A reversible wet/dry adhesive inspired by mussels and geckos. Nature.

[100] Dorrer C., Rühe J. (2008). Mimicking the Stenocara beetle: Dewetting of drops from a patterned superhydrophobic surface. Langmuir.

[101] Zheng Y., Bai H., Huang Z., Tian X., Nie F.Q., Zhao Y., Zhai J., Jiang L. (2010). Directional water collection on wetted spider silk. Nature.

[102] Claverie M., McReynolds C., Petitpas A., Thomas M., Fernandes S.C.M. (2020). Marine-derived polymeric materials and biomimetics: An overview. Polymers.

[103] Hennebert E., Maldonado B., Ladurner P., Flammang P., Santos R. (2015). Experimental strategies for the identification and characterization of adhesive proteins in animals: A review. Interface Focus.

[104] Mueller W.E.G., Wang X., Proksch P., Perry C.C., Osinga R., Garderes J., Schroeder H.C. (2013). Principles of biofouling protection in marine sponges: A model for the design of novel biomimetic and bio-inspired coatings in the marine environment?. Mar. Biotechnol..

[105] Picardi G., Chellapurath M., Iacoponi S., Stefanni S., Laschi S., Calisti M. (2020). Bioinspired underwater legged robot for seabed exploration with low environmental disturbance. Sci. Robot..

[106] Fish F.E. (2020). Advantages of aquatic animals as models for bio-inspired drones over present AUV technology. Bioinspir. Biomim..

[107] Bicchi A., Kumar V. Robotic grasping and contact: A review. Proceedings of the 2000 ICRA Millennium Conference, IEEE International Conference on Robotics and Automation, Symposia Proceedings Cat. No. 00CH37065.

[108] Hughes J., Culha U., Giardina F., Guenther F., Rosendo A., Lida F. (2016). Soft manipulators and grippers: A review. Front. Robot. AI.

[109] Bicchi A., Gabiccini M., Santello M. (2011). Modelling natural and artificial hands with synergies. Philos. Trans. R. Soc. B.

[110] Controzzi M., Cipriani C., Carrozza M.C., Balasubramanian R., Santos V. (2014). Design of artificial hands: A review. The Human Hand as an Inspiration for Robot Hand Development.

[111] Deimel R., Brock O. (2016). A novel type of compliant and underactuated robotic hand for dexterous grasping. Int. J. Robot. Res..

[112] Darus M.I.Z., Al-Khafaji A.A.M., Peng S.L., Favorskaya M., Chao H.C. (2021). Bio-inspired algorithms for modelling and control of underwater flexible single-link manipulator. Sensor Networks and Signal Processing, Proceedings of the 2nd Sensor Networks and Signal Processing (SNSP 2019), Hualien, Taiwan, 19–22 November 2019.

[113] Mura D., Barbarossa M., Dinuzzi G., Grioli G., Caiti A., Catalano M.G. (2018). A soft modular end effector for underwater manipulation: A gentle, adaptable grasp for the ocean depths. IEEE Robot. Autom. Mag..

[114] Galloway K.C., Becker K.P., Phillips B., Kirby J., Licht S., Tchernov D., Wood R.J., Gruber D.F. (2016). Soft robotic grippers for biological sampling on deep reefs. Soft Robot..

[115] Vogt D.M., Becker K.P., Phillips B.T., Graule M.A., Rotjan R.D., Shank T.M., Cordes E.E., Wood R.J., Gruber D.F. (2018). Shipboard design and fabrication of custom 3D-printed soft robotic manipulators for the investigation of delicate deep-sea organisms. PLoS ONE.

[116] Youssef I., Mutlu M., Bayat B., Crespi A., Hauser S., Conradt J., Bernardino A., Ijspeert A. (2020). A Neuro-inspired computational model for a visually guided robotic Lamprey using frame and event based cameras. IEEE Robot. Autom. Lett..

[117] Powell S.B., Garnett R., Marshall J., Rizk C., Gruev V. (2018). Bioinspired polarization vision enables underwater geolocalization. Sci. Adv..

[118] Dupeyroux J., Viollet S., Serres J.R. (2019). An ant-inspired celestial compass applied to autonomous outdoor robot navigation. Robot. Auton. Syst..

[119] Gul J.Z., Sajid M., Rehman M.M., Siddiqui G.U., Shah I., Kim K.H., Lee J.W., Choi K.H. (2018). 3D printing for soft robotics—A review. Sci. Technol. Adv. Mater..

[120] Gottwald M., Herzog H., Von Der Emde G. (2019). A bio-inspired electric camera for short-range object inspection in murky waters. Bioinspir. Biomim..

[121] Parrott C., Dodd T.J., Boxall J., Horoshenkov K. (2020). Simulation of the behaviour of biologically-inspired swarm robots for the autonomous inspection of buried pipes. Tunn. Undergr. Space Technol..

[122] Vedachalam N., Ramesh R., Jyothi V.B.N., Prakash V.D., Ramadass G.A., Atmanand M.A. (2020). Design considerations for strategic autonomous underwater swarm robotic systems. Mar. Technol. Soc. J..

[123] Praczyk T. (2020). Neural collision avoidance system for biomimetic autonomous underwater vehicle. Soft Comput..

[124] Zhu D., Cao X., Sun B., Luo C. (2018). Biologically inspired self-organizing map applied to task assignment and path planning of an AUV system. IEEE Trans. Cogn. Develop. Syst..

[125] Fernandez-Leon J.A., Acosta G.G., Rozenfeld A. (2014). How simple autonomous decisions evolve into robust behaviours? A review from neurorobotics, cognitive, self-organized and artificial immune systems fields. BioSystems.

[126] Boulogne L.H., Wolf B.J., Wiering M.A., Van Netten S.M. (2017). Performance of neural networks for localizing moving objects with an artificial lateral line. Bioinspir. Biomim..

[127] Szymak P., Piskur P., Naus K. (2020). The effectiveness of using a pretrained deep learning neural networks for object classification in underwater video. Remote Sens..

[128] Aiello B.R., Hardy A.R., Westneat M.W., Hale M.E. (2018). Fins as mechanosensors for movement and touch-related behaviors. Integr. Comp. Biol..

[129] Chen X.X., Huang J. (2019). Odour source localization algorithms on mobile robots: A review and future outlook. Robot. Auton. Syst..

[130] Leadbetter A.M., Shepherd A., Arko R., Chandler C., Chen Y., Dockery N., Ferreira R., Fu L., Thomas R., West P. (2016). Experiences of a “semantics smackdown”. Earth Sci. Inform..

[131] DiGiusepe N., Pouchard L.C., Noy N.F. (2014). SWEET ontology coverage for earth system sciences. Earth Sci. Inform..

[132] Edwards C. (2021). The best of NLP. Commun ACM..

[133] Van Dinter R., Tekinerdogan B., Catal C. (2021). Automation of systematic literature reviews: A systematic literature review. Inf. Softw. Technol..

[134] Van Raan A.F., Blockmans W., Engwall L., Weaire D. (2014). Advances in bibliometric analysis: Research performance assessment and science mapping. Bibliometrics. Use and Abuse in the Review of Research Performance. Wenner-Gren International Series.

[135] Ioannoni V., Vitale T., Costa C., Elliot I. (2020). Depicting communities of Romani studies: On the who, when and where of Roma related scientific publications. Scientometrics.

